# Solomon Islands Oncology Unit: Sustainability in Terms of Outcomes

**DOI:** 10.1200/GO.23.00325

**Published:** 2024-01-25

**Authors:** Dylan Bush, Mark Love, Hugo Bugoro, Nixon Panda

**Affiliations:** Dylan Bush, BA, Warren Alpert Medical School of Brown University, Providence, RI; Mark Love, PhD, Griffith University, Brisbane, Australia; and Hugo Bugoro, PhD and Nixon Panda, MAE, Solomon Islands National University, Honiara, Solomon Islands

## Abstract

Bush et al emphasize that the key to establishing enduring and efficient global health systems lies in prioritizing local stakeholders and, above all, the welfare of patients.

## TO THE EDITOR:

A recent article reported on the establishment of the first oncology unit at the National Referral Hospital (NRH) in Solomon Islands.^1^ This project was accomplished with support from the Australian Government Department of Foreign Affairs and Trade. In their article, the authors write that Solomon Islands now has a sustainable oncology unit delivering chemotherapy treatments.

Yip et al^1^ detail the efforts of Australian and Solomon Islands medical staff to improve oncology services. We applaud the establishment of the oncology unit at the NRH. However, we take this as a heuristic opportunity to raise and reiterate the ongoing critical challenge associated with the sustainability of donor-supported international health initiatives.

In their article, the authors do not demonstrate how the new oncology unit has affected clinical outcomes beyond increasing case load. Moreover, it is unclear what the authors mean by sustainability. Recent reports from the head of the NRH oncology unit highlight continued shortages of medication, space, and manpower that negatively affect clinical care.^2^ Thus we ask, how is this an example of a sustainable project?

Although debate continues about what sustainability means in global health, patient outcomes are an important measure of success and a definitive component of sustainability.^3^ Additionally, outcomes and reductions in health inequities—which require ongoing monitoring of clinical outcomes—must continue to improve after outside support decreases or ceases.^4^

A recent study by Cancedda et al^3^ argues that an outcome-focused, tangible, dynamic, and integrated approach is key to achieving sustainability in global health. Such an approach requires international funders and partners to provide longer-term and more integrated investments that allow for programmatic flexibility and adaptation to the local context. The WHO's recent Health Systems Resilience Toolkit defines resilient health systems in part by their ability to bring about positive health outcomes.^5^

Although the training of local staff and the creation of a dedicated oncology unit are important accomplishments, they do little to improve patient outcomes if chemotherapeutic agents are unavailable. Although the authors mention a 2018 visit to the Solomon Islands National Medical Store to discuss issues with medication supply chain, they do not explain what, if any, efforts were made to resolve or alleviate these deficits.

Furthermore, while the authors point to an increased case load (39 new cases in 2019, 99 new cases in 2023) as evidence of the program's success, it is unknown whether these patients received a full, uninterrupted course of chemotherapy. Existing research suggests a strong link between uninterrupted anticancer therapy and response to therapy.^6-9^ Reports from the oncology unit indicate that interruptions in chemotherapy are commonplace, with patients waiting up to 3 months to reinitiate therapy because of drug shortages.^1^

The establishment of an oncology unit is an important first step in improving cancer treatment in Solomon Islands. To make oncological services more sustainable, further efforts should be made to improve the provision of chemotherapeutic medications and monitoring of clinical outcomes. This requires continued international financial support and a more integrated, adaptive, longer-term approach. Only then will the goal of a resilient and sustainable health system in Solomon Islands manifest.
